# Prediction of the surface roughness of Ti-6Al-4 V alloy during surface grinding using machine learning models

**DOI:** 10.1038/s41598-026-53166-3

**Published:** 2026-06-01

**Authors:** K. Siri Varsha Reddy, B. Adithi, R. Nirupashree, S. Hari Lokitha, T. Satish Kumar, Ranjan Kumar Ghadai, Kanak Kalita

**Affiliations:** 1 Department of Mechanical Engineering, Amrita School of Engineering, Amrita Vishwa Vidyapeetham, Coimbatore, 641112 India; 2 Manipal Institute of Technology, Manipal Academy of Higher Education, Manipal, India; 3 Department of Mechanical Engineering, Rajalakshmi Institute of Technology, Chennai, 600124 India

**Keywords:** Ti-6Al-4V alloy, Surface roughness, Surface grinding, Abrasive wheel type, Machine learning, XGBoost, Engineering, Materials science

## Abstract

This study presents a machine learning (ML)-based approach to predict surface roughness, R_a_ during dry grinding of Ti-6Al-4 V alloy using Decision Tree, Random Forest, Gradient Boosting, Extreme Gradient Boosting (XGBoost), Linear Regression, and Polynomial Regression models. Experiments were conducted with Aluminium Oxide (Al₂O₃) and Silicon Carbide (SiC) wheels at varying feed rates (0.2–0.9 mm/rev) and depths of cut (0.02–0.08 mm). Results showed that Al₂O₃ consistently produced lower R_a_ values.Increased feed and depth of cut led to rougher surfaces. XGBoost algorithm achieved the highest prediction accuracy (R² = 0.90), effectively capturing nonlinear dependencies. Feature importance analysis identified feed rate as the most influential factor (Importance Score > 0.85), followed by depth of cut and wheel type. These findings demonstrate the potential of ML, particularly XGBoost, for optimizing grinding parameters and enhancing surface quality in Ti-6Al-4 V machining.

**Supplementary Information:**

The online version contains supplementary material available at 10.1038/s41598-026-53166-3.

## Introduction

Ti-6Al-4 V is one of the most commonly used titanium alloys in the aerospace, biomedical, and automotive industries as it has a great mix of mechanical properties, including high specific strength, corrosion resistance, and low density. It is a great choice for important engineering uses because it can retain its strength at high temperatures and resist deformation under load^1,2^. However, these inherent properties that make Ti-6Al-4 V an exceptional material also pose some significant challenges during grinding. Its high strength, low elastic modulus, low thermal conductivity, and tendency for work hardening collectively contribute to the increased difficulty in grinding this alloy efficiently^3^. During grinding or machining, localized temperatures rise rapidly, promoting interactions between the alloy surface and atmospheric gases such as oxygen, nitrogen, and hydrogen. This not only weakens the surface ductility, but it also makes it more brittle, which hurts performance in applications that are prone to fatigue^4^.

Despite machining difficulties, Ti-6Al-4 V is extensively used for components like aerospace parts, biomedical implants, and high-performance engine parts. Hence, the surface roughness produced by this alloy plays a significant role, as it effects the dimensional accuracy of the manufactured components^5^. Surface roughness is a key indicator of surface integrity. It describes the microscopic irregularities present on a machined surface. It impacts functional properties like fatigue life, wear resistance, tribological behaviour, and residual stress. So, optimal surface finish ensures maximum product life.

Recent studies have utilized Response Surface Methodology (RSM)^6^, Adaptive Neuro-Fuzzy Inference System (ANFIS) and Multiple Regression Analysis (MRA)^7^ for predicting cutting forces, surface roughness, and tool wear during machining. Machine Learning (ML) methods are also employed to predict machining responses^8^. Digital manufacturing and smart machining have made significant progress in the past few years^9^. Currently, artificial intelligence (AI) and Machine Learning (ML) methods are being utilized to model and optimize complex machining operations. These tools help make predictions about process behaviours, which reduces reliance on trial and error. Several researchers have used machine learning methods to predict the wear rate of grinding wheel and the grinding force it requires, to monitor the condition of the wheel, to find grinding burns, and to predict surface roughness^10–19^. For instance, Nosenko et al.^11^ investigated the effect of dressing the tools and changing the feed direction on the deep grinding of titanium alloys with silicon carbide wheels. The importance of conditioning of the grinding wheels to achieve the optimal surface quality was highlighted. Guo et al.^12^ demonstrated that alteration of feed rate and depth of cut significantly influences the surface finish and energy efficiency during the SiC based grinding of Ti-6Al-4 V.

Manufacturing systems, everyday, are increasingly turning to machine learning to analyse sensor data in real time, as it allows for adaptive process control. Nguyen et al.^20^ integrated ML, Multi-Objective Optimization (MOO), and Multi-Criteria Decision Making (MCDM) to optimize milling conditions for AISI 4140 steel. Four ML models were used: XGBoost, CatBoost, Gradient Boost, and LightGB to predict surface roughness (Ra), tool wear (Vb), cutting force (Fc), and material removal rate (MRR).The results indicated that different models were better at predicting different performance outcomes.

Siyambas et al.^21^ used ensemble ML algorithms, such as Random Forest and enhanced versions of XGBoost, to predict the surface roughness of Ti-6Al-4 V during turning under different cooling conditions. The optimized model had a high R² of 0.9914, which indicates machine learning’s capability to make accurate predictions. Mazid et al.^22^ used SVM, Random Forest, and deep learning algorithms to model the effects of turning parameters, tool geometry, and coatings on surface finish. The SVM model with RBF kernel had the best predictive accuracy, showing machine learning’s capability to capture nonlinear relationships between process parameters and surface roughness during machining of titanium.

Further, Prashanth et al.^23^ examined the influence of process parameters on grinding of Inconel 751 alloy under different environmental conditions.ML models: SVM, Gaussian Process Regression (GPR), and Boosted Tree Ensemble, were used to develop the predictive models which were analyzed using K-fold cross validation technique. Performance was evaluated using R² and RMSE, with GPR showing the best predictive results. Anand et al.^24^ implemented several machine learning algorithms to investigate the grinding performance of additively manufactured Ti-6Al-4 V under different cooling conditions. The study found Random Forest as most effective algorithm, achieving the lowest MSE (0.000178) in surface roughness prediction.

While numerous studies have explored the role of machine learning in modelling surface integrity for difficult-to-machine alloys, relatively limited attention has been paid to the combined influence of abrasive type, feed rate and depth of cut on the surface roughness of Ti-6Al-4 V during dry grinding. Moreover, previous works have often relied on turning or milling processes rather than surface grinding, which involves different tool – workpiece interaction dynamics. The present study aims to fill this critical gap by developing robust ML based models capable of predicting surface roughness in Ti-6Al-4 V grinding using key input variables – abrasive wheel type, feed rate and depth of cut. Hence, this work contributes to the advancement of intelligent process modelling for high-performance titanium alloy machining and supports the ongoing transition toward data-driven manufacturing ecosystems. Novelty of this work specifically, is the integration of abrasive type, feed rate, and depth of cut in dry grinding of Ti-6Al-4 V alloy, coupled with predictive machine learning models.

## Materials and methods

### Material

The material chosen for this study is Ti-6Al-4 V alloy, which is a dual-phase (α + β) titanium alloy. Ti-6Al-4 V comprises 6% aluminium, an α-phase stabilizer that enhances oxidation resistance and contributes to both low- and high-temperature strength and 4% vanadium, a β-phase stabilizer that provides moderate solid solution strengthening. These alloying elements collectively impart high strength, excellent corrosion resistance, and good formability. Its primary chemical composition is presented in Table 1^24^, while Table 2 summarizes its key mechanical and physical properties. For the present study, the Ti-6Al-4 V alloy was cast as a single sample and machined by Electrical Discharge Machining (EDM) to samples of size: 50 mm × 30 mm × 12 mm.

**Table 1:** Composition of Ti-6Al-4 V alloy (wt%).

Element	Al	V	Fe	C	*N*	H	O	Ti
Wt. %	5.5–6.5	3.5–4.5	0.3	0.1	0.05	0.015	0.2	Remaining

**Table 2:** Mechanical properties of Ti-6Al-4 V alloy.

Property	Value	Property	Value
Yield Strength (MPa)	830–900	Density (g/cm^3^)	4.41–4.43
Ultimate tensile Strength (MPa)	900–980	Toughness (MJ/m^3^)	78–100
Elongation (%)	10–15	Thermal Conductivity (W/m-K)	6.7–7.0
Hardness (HV)	301–342	Thermal Expansion (10^− 6^/K)	8.6–9.6

### Methodology

**Fig. 1:**
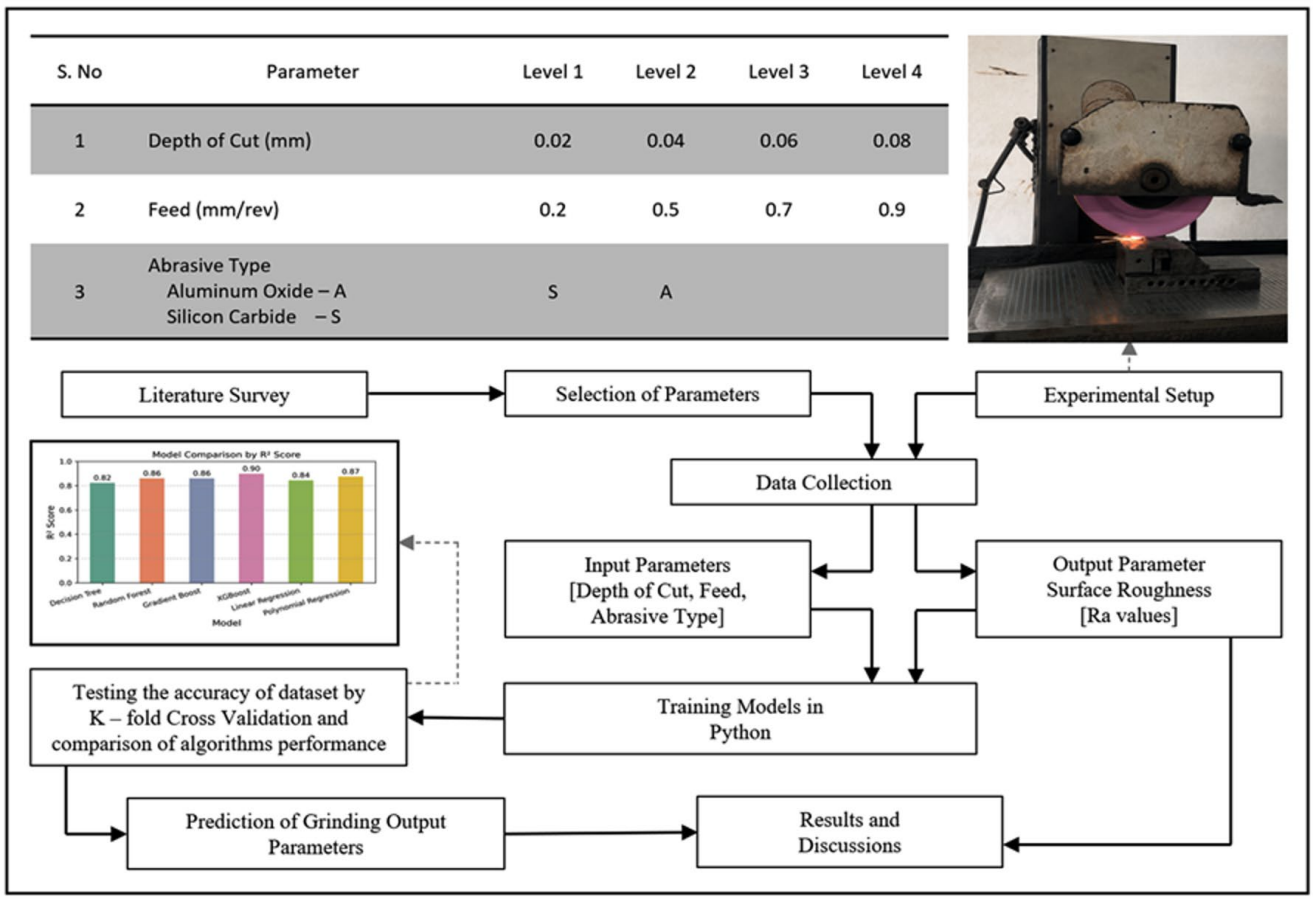
Methodology flowchart.

The experimental procedure and machine learning based prediction workflow are illustrated in Fig. 1, which shows the methodology flowchart for experimental grinding and surface roughness prediction in Ti-6Al-4 V alloy.

### Surface grinding and environmental conditions

The grinding operation was conducted using a horizontal spindle surface grinding machine, employing two distinct types of grinding wheels – aluminium oxide, and silicon carbide wheels. The grinding variables examined include the depth of cut (0.02 mm, 0.04 mm, 0.06 mm, 0.08 mm) and feed rate (0.2 mm/rev, 0.5 mm/rev, 0.7 mm/rev, 0.9 mm/rev).

**Fig. 2:**
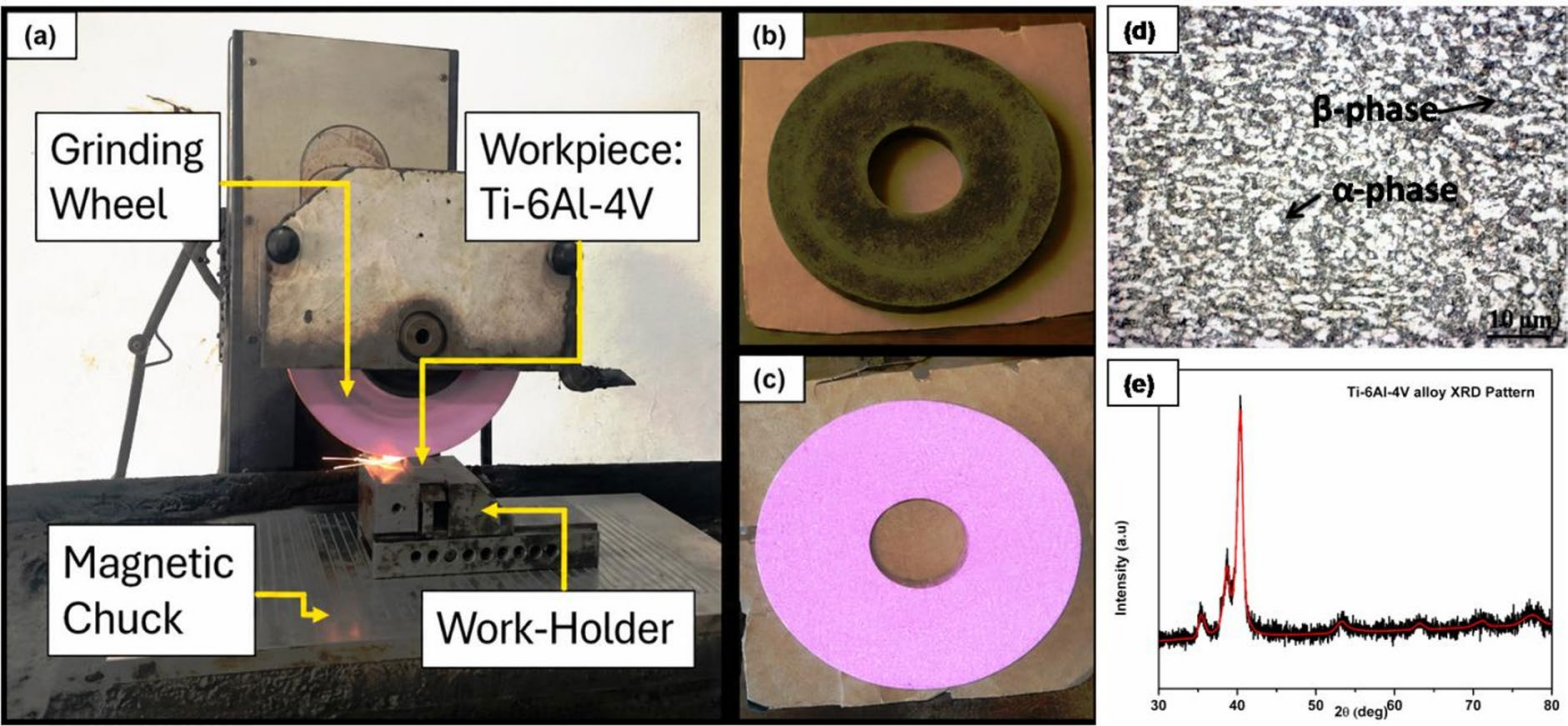
(**a**) Grinding Process, (**b**) Silicon Carbide Wheel, (**c**) Aluminium Oxide Wheel, (**d**) Microstructure of Ti-6Al-4 V alloy and (**e**) XRD pattern of Ti-6Al-4 V alloy.

The optical microstructure (Fig. 2d), and XRD pattern (Fig. 2e), for Ti-6Al-4 V alloy and gives some understanding about the phases present and structural features. First of all, it can be clearly seen that the alloy microstructure (Fig. 2d), contains two phases which are identified by the optical micrograph image as equiaxed α-phase with bright areas and β-phase with dark ones. Thus, there is the presence of the homogeneous distribution of the β-phase through the α -matrix, which is characteristic for wrought Ti-6Al-4 V and gives a contribution to balanced mechanical properties (strength and hardness), necessary for proper material removing and surface finishing during grinding. Also, from the XRD pattern (Fig. 2e), it is evident that both the mentioned phases are present – hexagonal close packed alpha phase and body centered cubic beta phase. Speaking about grinding from this point of view, one should notice that the co-existence of these two phases may have some effect on roughness value obtained because alpha-phase is much more difficult to remove because of higher hardness and the beta phase is easier due to higher deformation resistance.

**Table 3:** Grinding process parameters.

Parameter	Condition
Grinding Mode	Surface Type
Environment	Dry
Workpiece Material	Ti-6Al-4 V alloy
Wheel Speed	29.32 m/s
Feed Rate	0.2–0.9 mm/rev (0.56–2.52 m/min)
Depth of Cut	20–80 μm
Abrasive Type	Al_2_O_3_,SiC

**Table 4:** Specifications of Abrasive Wheels.

Specification	SiC	Al_2_O_3_
Wheel Number	C60 K5 V206	RAA60 K5 V206
Diameter	206 mm	206 mm
Grain Type	Black SiC	Pink Al_2_O_3_
Grain Size	60	60
Wheel Grade	K – Medium	K – Medium
Bond Type	Vitrified (V)	Vitrified (V)
Wheel Structure	5	5

The grinding operation employed is illustrated in Fig. 2 (a), while Figs. 2 (b) and 2(c) display the Silicon Carbide and Aluminium Oxide grinding wheels utilized during the experiments. The grinding process parameters and detailed specifications of the abrasive wheels are provided in Tables 3 and 4, respectively. The grinding machine used was Bhurji Machine Tools AH-630 Model (detailed specifications are available in Supplementary Material).A fresh grinding wheel was used for the experiments, and no dressing was performed between runs to ensure consistency under identical wheel conditions.

Surface roughness (Ra) was measured using the Mitutoyo Surftest SJ-410(detailed specifications are available in Supplementary Material). A cut-off length of 2.5 mm was selected, and the evaluation length was maintained at 12.5 mm (5 × cut-off length). Ten measurements were taken at different locations on each specimen, and the reported Ra value represents the average of these readings. Measurements were conducted parallel to the grinding marks to ensure consistency.

### Machine learning

#### Dataset

Tables 5, 6 and 7.

**Table 5:** Sample dataset.

Feed (mm/rev)	Depth of Cut (mm)	Wheel Type	Ra (µm)
0.2	0.02	1	0.154
0.2	0.04	1	0.162
0.2	0.06	1	0.194
0.2	0.08	1	0.203

**Table 6:** Dataset information.

Criterion	Details
Number of Instances	32 (each mean of 3 repeated experiments)
Number of attributes	3
Number of classes	1 (Continuous Type)

**Table 7:** Descriptive statistics for surface roughness (Ra).

Feature	Value (µm)
Minimum	0.151
Maximum	0.318
Mean	0.226
Standard Deviation	0.05

#### Machine learning models

##### Decision tree

The Decision Tree Regressor was employed as a simple nonlinear machine learning baseline model due to its interpretability and straightforward implementation. This model partitions the input feature space into hierarchical subsets based on threshold values, resulting in a tree-structured representation. Each terminal (leaf) node corresponds to a predicted surface roughness value. By minimizing the variance within each node during the splitting process, the model effectively captures nonlinear dependencies between input parameters and the target variable, without relying on any prior assumptions regarding data distribution.

##### Random forest

Random Forest improves the decision tree method by combining predictions from several trees that were built on randomly chosen data and features. This ensemble method makes generalization better and overfitting less likely. In this study, unrestricted tree depth and no feature selection, yielded robust predictions of surface roughness under varied grinding conditions.

##### Gradient boost

Was employed to iteratively construct an ensemble of decision trees, where each successive tree aimed to minimize the residual errors of its predecessors. The model was trained using 100 estimators with default hyperparameters, and a fixed random seed of 42 was applied to ensure consistency and reproducibility across experimental runs.

##### XG Boost

XGBoost is a scalable and efficient implementation of gradient boosting that incorporates regularization to control overfitting. In this work, it was applied with standard parameters to predict surface roughness. Its performance reflects the model’s strength in handling nonlinearities, feature interactions, and high-dimensional data, while maintaining computational efficiency.

##### Linear regression

Linear Regression was used to model the relationship between grinding parameters and surface roughness under the assumption of linear dependence. Despite its simplicity, it provided a useful benchmark for comparison, although its predictive power is limited in the presence of nonlinear interactions. Therefore, it served as the fundamental statistical baseline.

##### Polynomial regression

Polynomial Regression extends linear regression by introducing non-linear terms, enabling the model to fit complex curves. A degree-3 polynomial was used to capture higher-order interactions among the input variables. The model was implemented using polynomial feature transformation followed by linear regression on the expanded feature set.

#### Performance metrics

The following performance metrics were employed for a thorough assessment of model performance, to ensure surface roughness can be accurately predicted based on the grinding parameters.

Let n denote the number of instances, $\:{y}_{i}$ signify the empirically measured output value, $\:{\widehat{y}}_{i}$ signify the predicted output value and $\:\stackrel{-}{y}$ represent the arithmetic mean of empirically measure output values.

##### Coefficient of determination (R²)

The proportion of variability in the response variable that is explained by the regression model relative to a constant-mean baseline. It equals 1 for perfect predictions, 0 when the model is no better than the sample-mean predictor, and can be negative if it performs worse; its interpretation assumes an intercept (or a centred response). It is defined as:

$$\:{R}^{2}\:=\:\frac{{\sum\:}_{i=1}^{n}{(\:{y}_{i}\:-\:{\widehat{y}}_{i}\:)}^{2}}{{\sum\:}_{i=1}^{n}{(\:{y}_{i}\:-\:\stackrel{-}{y}\:)}^{2}}$$

##### Mean squared error (MSE)

The average of the squared prediction errors, measuring the typical squared deviation between model outputs and observed values; it penalizes large errors more heavily and is expressed in squared units of the target. It is defined as:

$$\:MSE\:=\:\frac{1}{n}{\sum\:}_{i=1}^{n}{(\:{y}_{i}\:-\:{\widehat{y}}_{i}\:)}^{2}$$

##### Root mean squared error (RMSE)

The square root of the MSE, representing the typical magnitude of prediction error in the same units as the target; it is more sensitive to large errors and is often used for direct, engineeringscale interpretation. It is defined as:

$$\:RMSE\:=\:\sqrt{\frac{1}{n}{\sum\:}_{i=1}^{n}{(\:{y}_{i}\:-\:{\widehat{y}}_{i}\:)}^{2}}$$

##### Mean absolute error (MAE)

The average of the absolute prediction errors, giving a straightforward measure of typical error size in the target’s units; it applies a linear penalty to deviations and is less influenced by outliers than RMSE. It is defined as:

$$\:MAE\:=\:\frac{1}{n}{\sum\:}_{i=1}^{n}{\left|{y}_{i}\:-\:{\widehat{y}}_{i}\right|}^{2}$$

#### Model evaluation workflow

Model performance was evaluated using 5-fold cross-validation. The dataset was divided into five equal subsets; in each iteration, four subsets (80%) were used for training and the remaining subset (20%) for testing. This procedure was repeated five times so that each data point served once as test data. All the models were evaluated using this approach. Performance metrics (R², MSE, RMSE, and MAE) were computed for each fold and averaged to obtain the final model performance. The complete list of model hyperparameters is provided in the supplementary material. No hyperparameter tuning was performed, minimizing the risk of information leakage and making efficient use of the limited dataset. All the programming was performed in Jupyter Lab (Version 3.6.8).

##### Limitations

Given the small size of the dataset, no separate 80/20 hold-out test set was used. While 5-fold cross-validation provides robust performance estimates, the absence of an independent test set may limit the evaluation of generalization to entirely unseen data. Future studies with larger datasets could incorporate a hold-out test set to further validate model performance and strengthen confidence in the results.

## Results and discussions

### Analysis of surface roughness

#### Influence of abrasive type on surface roughness

**Fig. 3:**
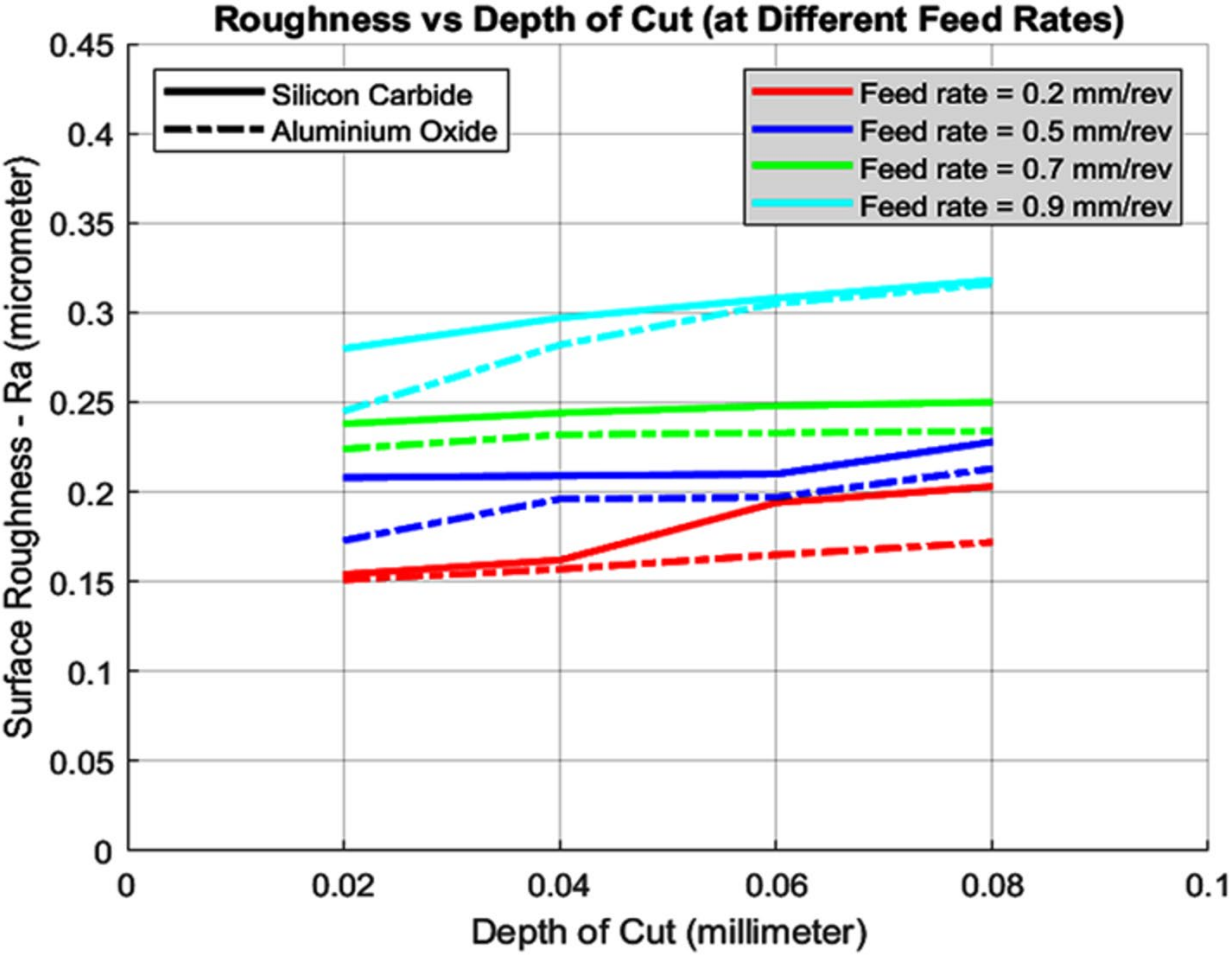
Surface Roughness vs. Depth of Cut (at different Feed rates for different abrasive wheels).

The experimental findings underscore the significant impact of abrasive wheel composition on the surface roughness of Ti-6Al-4 V alloy during surface grinding. As illustrated in Fig. 3, aluminium oxide (Al₂O₃) grinding wheels produced consistently lower average surface roughness values than silicon carbide (SiC) wheels. This performance advantage could likely be due to the superior friability of Al₂O₃ grains, which enables the continuous regeneration of sharp cutting edges, thereby minimizing friction, thermal loading, and surface defects. These observations are consistent with Mukhopadhyay and Kundu^25^, who demonstrated that Al₂O₃ wheels yield improved surface finishes for titanium alloys. Similarly, de Mello et al.^26^ reported that the higher hardness and thermal conductivity of Al₂O₃ abrasives facilitate efficient material removal with fewer surface irregularities, making them more suitable for grinding heat-sensitive alloys like Ti-6Al-4 V.

#### Effect of feed rate on surface roughness

An increase in feed rate was observed to correlate positively with surface roughness. This trend suggests that higher feed rates introduce more aggressive interaction between the abrasive grains and the workpiece surface, resulting in elevated cutting forces and vibrations that compromise the surface finish, as observed in Fig. 3. Li and Wang^27^ similarly reported that higher feed rates during Ti-6Al-4 V machining increase the undeformed chip thickness, leading to intensified tool-workpiece engagement and surface irregularities. Furthermore, Safari et al.^28^ attributed this degradation in surface quality to increased tool wear and thermal loading under high feed conditions. These factors collectively contribute to higher Ra values at elevated feed rates.

#### Influence of depth of cut on surface roughness

The analysis reveals that increasing the depth of cut leads to higher surface roughness values. This behaviour can be attributed to deeper tool penetration, which generates higher cutting forces and a larger contact area between the abrasive and the workpiece. Consequently, the probability of material tearing, plastic deformation, and thermal-induced surface deterioration increases. These results, as shown in Fig. 3, align with the findings of Sun et al.^29^, who demonstrated that greater depths of cut during titanium alloy grinding exacerbate tool engagement and heat generation, adversely affecting surface integrity. Additionally, Li and Wang^27^ reported that increased depth of cut contributes to higher residual stresses and material deformation, which further compromise the surface finish of Ti-6Al-4 V component.

### Evaluation of machine learning model performance

Among the various machine learning algorithms tested, the Extreme Gradient Boosting (XG Boost) model exhibited superior performance, achieving an R² value of 0.898, a minimal mean squared error (MSE) of 0.000152, and the lowest mean absolute error (MAE) of 0.00887 μm, as detailed in Table 8. These metrics indicate a high level of predictive accuracy and strong agreement between the predicted and actual surface roughness values.

**Table 8:** Comparison of performance metrics values for different models.

Model	*R*^2^ Score	MSE	RMSE	MAE
Decision Tree	0.8239	0.000273	0.015961	0.011895
Random Forest	0.8602	0.000210	0.013932	0.011075
Gradient Boost	0.8597	0.000215	0.014047	0.010604
XG Boost	0.8982	0.000152	0.011941	0.008873
Linear Regression	0.8447	0.000235	0.015017	0.013020
Polynomial Regression	0.8744	0.000183	0.013317	0.010410

The superior performance of XGBoost can be attributed to its sequential boosting framework, which iteratively corrects residual errors from previous trees. Unlike a single Decision Tree, which partitions the feature space abruptly, XGBoost builds an ensemble of shallow trees that progressively refine predictions. This enables it to effectively capture nonlinear interactions between feed rate, depth of cut, and abrasive type without introducing excessive variance.

Polynomial Regression (R² = 0.874) also performed competitively, as it can model nonlinear relationships and interaction effects. However, polynomial models impose a global functional form on the data. In contrast, XGBoost adapts locally to variations in the feature space, allowing it to model complex, non-uniform relationships more flexibly.

Random Forest and Gradient Boosting also achieved good performance (R² > 0.85) by leveraging ensemble learning. However, Random Forest constructs trees independently and averages them, which may smooth predictions but does not iteratively correct errors. In contrast, XGBoost optimizes residuals sequentially, leading to improved bias reduction.

Decision Tree and Linear Regression showed comparatively lower accuracy with R² scores (0.824 and 0.845, respectively). Linear Regression is constrained by its linearity assumption and therefore cannot adequately represent curvature and interaction effects inherent in grinding processes. The Decision Tree, while nonlinear, suffers from high variance and discontinuous prediction boundaries, resulting in reduced generalization performance.

**Fig. 4:**
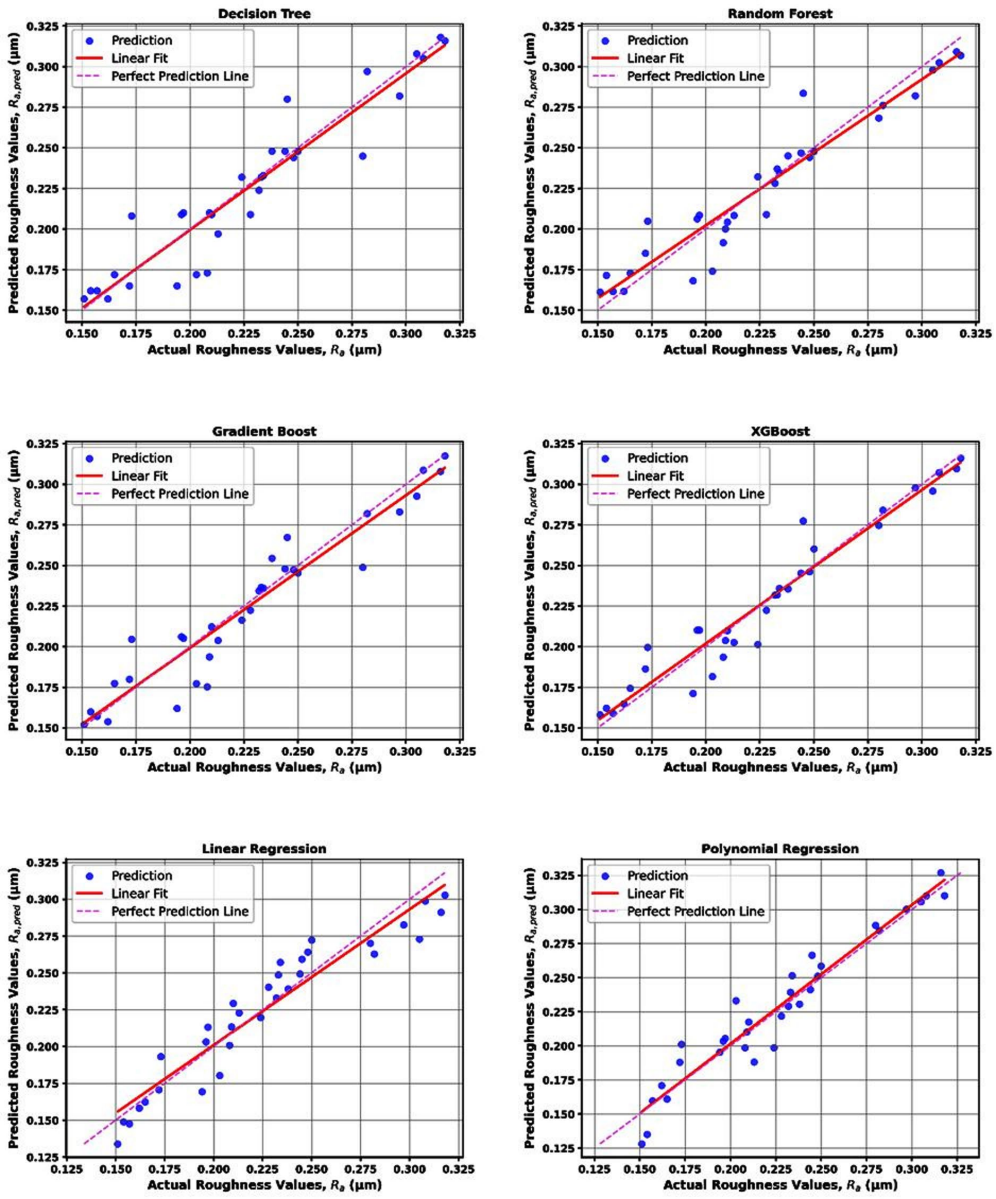
Relation between the actual and predicted surface roughness values for different models.

Figure 4 shows plots that compare actual surface roughness values to predicted values. These plots provide valuable insights into the performance of the evaluated machine learning models and their potential applicability to other contexts. A model’s prediction accuracy is reflected by the degree of alignment between the data points and the 45° ideal (perfect prediction) line. Among the six models considered, XG Boost exhibits the most precise predictions, with minimal deviation from the ideal line across the entire range of surface roughness values. This indicates that the model successfully captures the intricate nonlinear dependencies between the input process parameters – depth of cut, feed rate, and abrasive type – and the output Ra values.

The tight clustering suggests low residual error and strong generalization. These characteristics are particularly advantageous for deployment in real-time surface quality monitoring systems in advanced manufacturing. Polynomial Regression also performs commendably, closely tracking the ideal line. The model effectively captures the curvilinear relationship between input features and surface roughness, particularly in mid-range Ra values. However, slight deviations at the extreme ends of the roughness range suggest that polynomial models, while capturing trends, may require careful tuning of the polynomial order to avoid overfitting or underfitting. In contrast, Decision Tree and Linear Regression models show substantial scatter, especially at the boundaries. For Decision Tree, the lack of smoothing between decision boundaries results in step-like, discontinuous predictions that compromise accuracy. Linear Regression, constrained by its linearity assumption, fails to capture the curvature and interaction effects among input parameters, leading to systematic underestimation at high Ra values and overestimation at low values.

**Fig. 5:**
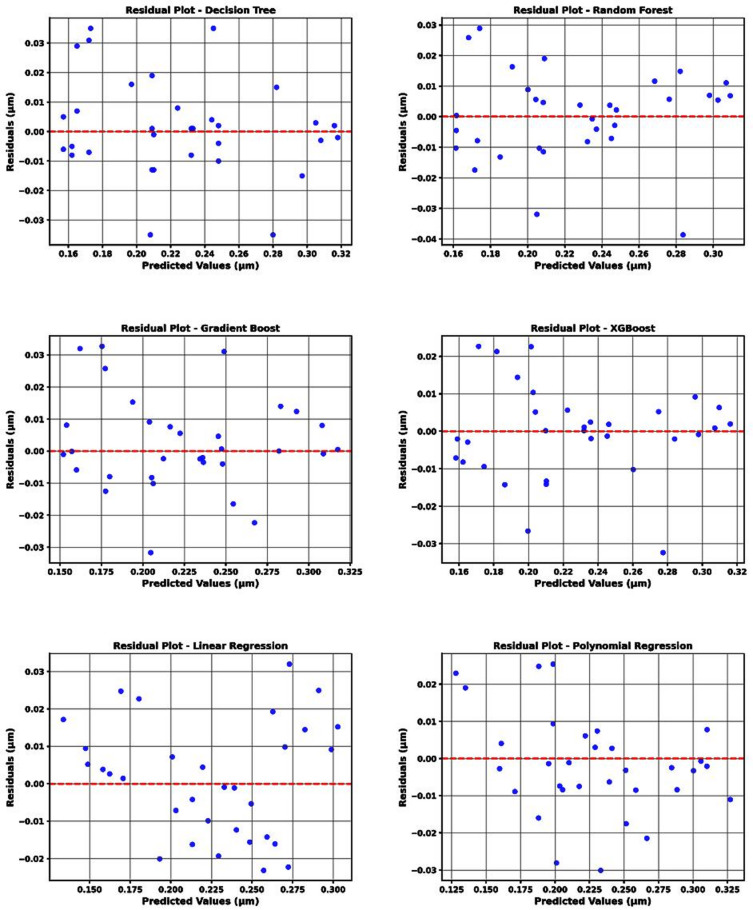
Residual Plots for different ML Models for prediction of Surface Roughness.

To complement the performance metrics and the predicted vs. actual plots, residual plots were prepared for all six models (Fig. 5). The residual plots depict the difference between predicted and measured Ra values across the dataset. Ideally, residuals should be randomly scattered around zero without discernible patterns.

For XGBoost, residuals remain tightly clustered around zero, indicating consistent predictive performance and absence of systematic bias. Importantly, no visible pattern is observed, suggesting stable variance throughout the response range. Polynomial Regression also demonstrates relatively small residual dispersion from the mid-range of Ra values; however, slight systematic deviations are noticeable at lower roughness levels. This indicates that while the polynomial model captures overall curvature, it struggles to fully represent localized nonlinear behaviour at the extremes.

Linear Regression exhibits clear structured patterns in the residuals. At lower and higher Ra values, the model tends to overpredict, while at mid-range Ra values, it underpredicts, indicating model bias due to its linear assumption. The Decision Tree model shows scattered but discontinuous residual behaviour, with larger deviations concentrated at specific Ra intervals, reflecting high variance and abrupt partitioning of the feature space.Random Forest and Gradient Boosting reduce this instability compared to a single Decision Tree, yet moderate dispersion remains, particularly at lower Ra levels.

Overall, the residual distribution confirms that XGBoost maintains low bias and controlled variance across all surface roughness ranges, resulting in more reliable and consistent predictions under varying grinding conditions. These findings reinforce the conclusions drawn from the Table 8; Fig. 4. They underscore the importance of model selection in data driven manufacturing applications and point toward the increasing relevance of advanced ensemble and non-linear models in predictive process optimization.

**Fig. 6:**
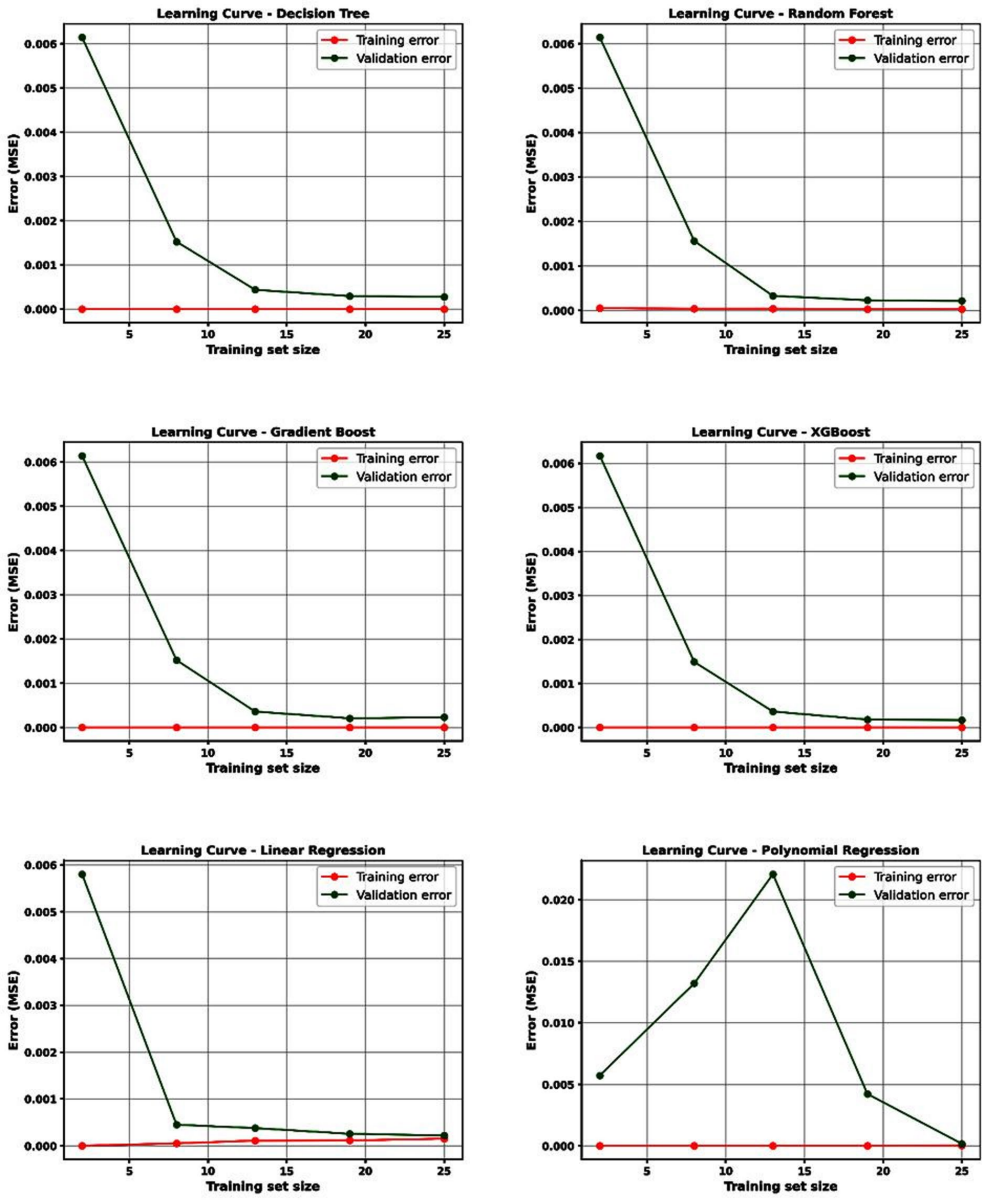
Learning curves for different ML models.

Learning curves were plotted for all six models to evaluate their learning behaviour and generalization capability as a function of training sample size (Fig. 6). For XG Boost, Random Forest, and Gradient Boost, both training and validation errors decrease progressively with increasing data and converge toward low, stable values. The small gap between the two curves indicates good generalization performance with minimal overfitting, demonstrating that these ensemble models effectively capture the underlying nonlinear relationships governing surface roughness.

In contrast, the Decision Tree model exhibits a significant gap between training and validation errors. The training error remains very low while the validation error stabilizes at a higher level than other models. Linear Regression shows relatively high training and validation errors that converge early, suggesting high bias and underfitting, as the linear structure fails to represent the nonlinear interactions between grinding parameters and surface roughness.

Polynomial Regression demonstrates fluctuating validation error at intermediate training sizes, reflecting sensitivity to sample size and possible instability in coefficient estimation. Although its final performance improves with more data, the temporary divergence between curves indicates moderate variance behaviour.

Overall, the learning curve analysis confirms that XG Boost achieves the most balanced bias–variance trade-off, combining low error with stable convergence. The ensemble-based models consistently outperform simpler parametric approaches, highlighting the importance of model complexity and adaptive learning mechanisms in accurately predicting surface roughness in grinding processes.

#### Comparison of model performance

**Fig. 7:**
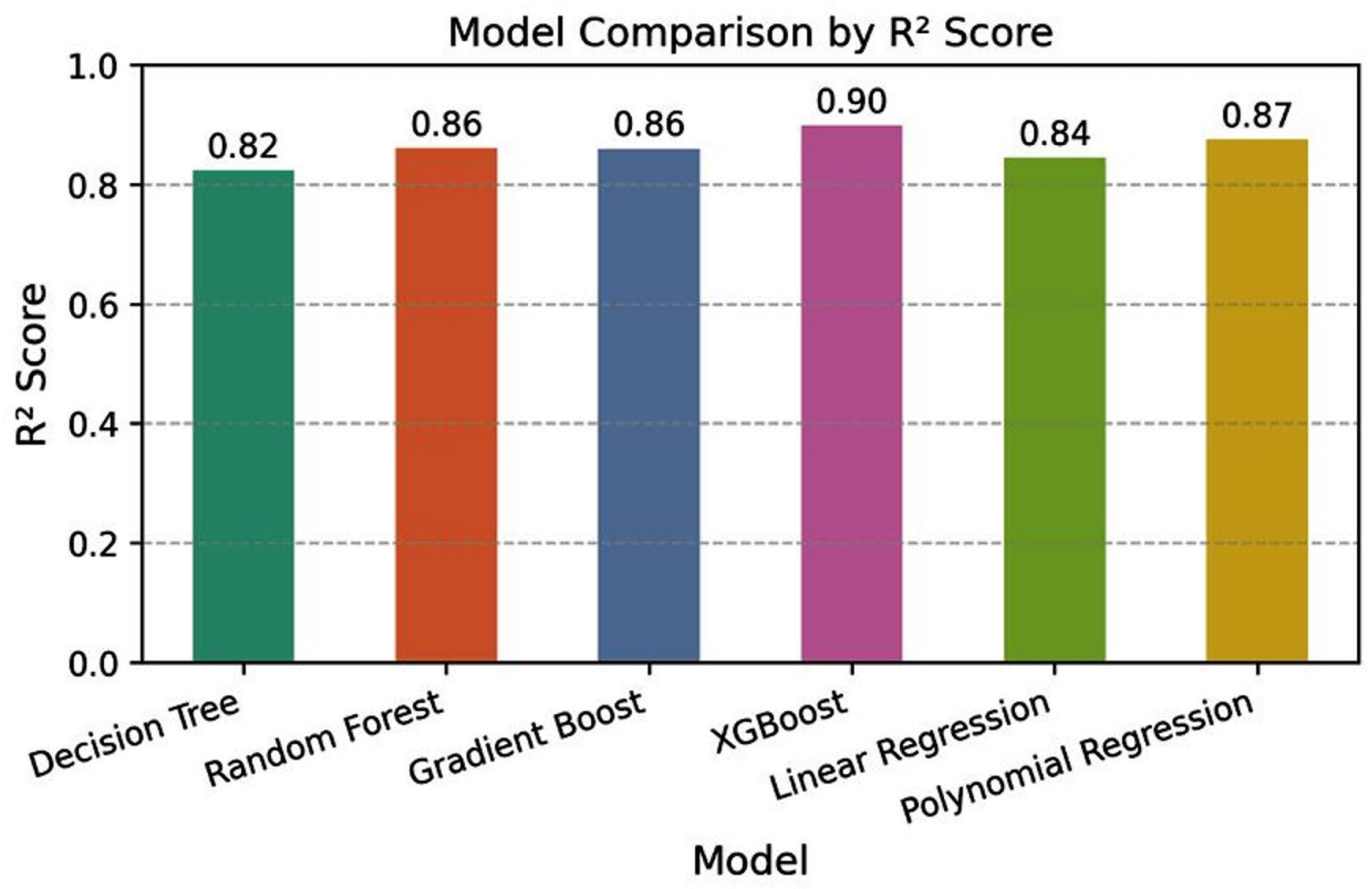
Comparison of R² scores for different machine learning models used to predict surface roughness.

The bar chart presented in Fig. 7 reveals the R² score comparison for all six different machine learning models employed to predict surface roughness (R_a_) of Ti-6Al-4 V during grinding. Among the tested models, XG Boost achieved the highest R² score of 0.90, highlighting its superior ability to learn complex nonlinear relationships and deliver highly accurate predictions. This performance aligns with its earlier validation in Fig. 4, where predicted values tightly clustered around the perfect prediction line.

Polynomial Regression and Gradient Boosting also demonstrated commendable performance, with R² scores of 0.87 and 0.86, respectively. These models effectively capture the nonlinear patterns between process variables and surface roughness. Random Forest followed closely with an R² of 0.86, reflecting the stability and robustness provided by ensemble averaging techniques. On the other hand, Linear Regression yielded an R² score of 0.84, indicating moderate predictive capability. However, its inability to handle interactions and nonlinear effects limits its effectiveness in precision manufacturing contexts. Decision Tree, with the lowest R² value of 0.82, underperformed due to its susceptibility to overfitting and lack of smoothing between decision thresholds. These results reaffirm that ensemble-based and non-linear models are better suited for modelling grinding operations where surface quality is influenced by complex interactions between multiple process parameters.

**Table 9:** Summary of ML based surface roughness prediction studies.

Study	Material	ML models	Best model & metrics
Siyambas et al^21^.	Ti-6Al-4 V	Random Forest Ada Boost XG Boost Cat Boost	XG Boost R^2^ = 0.9914 and MAPE = 6.73
Prashanth et al^23^.	Inconel 751	SVM GPR Boosted Tree Ensemble	SVM R^2^ = 0.82 and RMSE = 0.0433
Anand et al^24^.	Ti-6Al-4 V	Gradient XG Boost Random Forest Neural Network	XG Boost R^2^ = 0.9789 and MSE = 9.5201
Ramdev et al.^30^	Inconel 800	linear regression, ridge regression, and random forest	Random Forest R^2^ = 0.935

Table 9 provides a comparative overview of recent machine learning approaches applied to surface roughness prediction across various materials and modelling techniques. Notably, gradient boosting based algorithms, particularly XG Boost, consistently emerge as top performing models, delivering superior prediction accuracy across multiple studies. For instance, Siyambas et al.^21^ reported an R^2^ value of 0.9914, while Anand et al.^24^ achieved R^2^ = 0.9789 using the same model. These findings underscore the robustness and adaptability of XG Boost in capturing complex nonlinear relationships influencing surface quality. In alignment with these results, the present study also identified XG Boost as the most effective model for prediction of Ra values in Ti-6Al-4 V, attaining a high coefficient of determination (R^2^ = 0.9), thereby reinforcing its suitability for accurate surface roughness prediction in machining applications.

#### Feature importance analysis

**Fig. 8:**
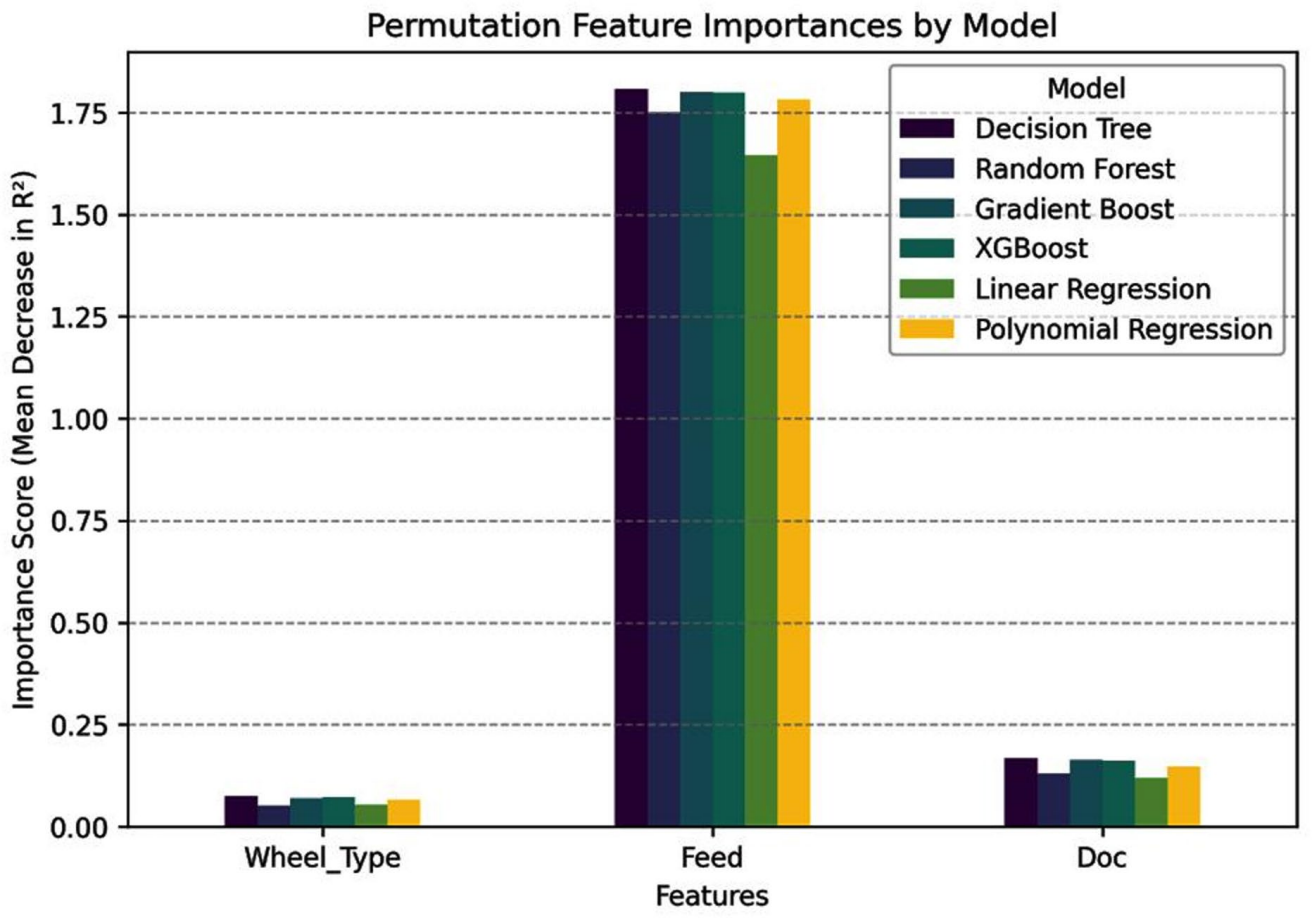
Feature importance of input parameters (Wheel Type, Feed, and Depth of Cut).

The feature importance analysis shown in Fig. 8 provides insight into how the three input parameters – Abrasive Wheel Type, Feed, and Depth of Cut (DoC) – influence model predictions across multiple algorithms. The most dominant feature across all models is Feed, with importance scores consistently exceeding 1.7 in the permutation tests. This underscores the critical role of feed rate in determining surface finish, which is consistent with the experimental observations discussed earlier. Higher feed rates are known to increase chip thickness, cutting forces, and vibration, all of which directly contribute to increased surface roughness.

Depth of Cut ranks as the second most important parameter, contributing moderately to prediction accuracy across models. This finding aligns with the understanding that deeper cuts increase tool–workpiece contact area and thermal load, thereby influencing surface roughness values.

Abrasive Wheel Type registers the lowest importance scores in the model-based analysis. Nonetheless, it should not be overlooked. While its statistical contribution is relatively smaller than feed and DoC, experimental trends confirm that wheel type does have a meaningful influence on surface finish, particularly due to differences in wheel material and structure that alter the grinding interaction. From a practical standpoint, these results hold direct value for process optimization. Feed rate and depth of cut are the most effective parameters to regulate surface roughness, as they can be adjusted more easily during operation. Wheel type, though less flexible to change, still plays an important role, especially when considering material-specific grinding requirements.

## Conclusion

This study used two abrasive wheel types – silicon carbide (SiC) and aluminium oxide (Al_2_O_3_) – to assess the surface grinding performance of Ti-6Al-4 V alloy under dry conditions both experimentally and through machine learning-based modelling. The following is a summary of the main conclusions drawn from the experimental trials and predictive analyses:

1. The Al₂O₃ grinding wheel consistently produced smoother surfaces than the SiC wheel, establishing its effectiveness in achieving improved surface quality when machining titanium alloys.
2. It was found that increasing feed rates and depths of cut significantly increased surface roughness (Ra). Higher cutting forces, thicker chips, and higher thermal loads are the causes of this trend, which emphasizes the necessity of exact control over input parameters in grinding applications.
3. With an R^2^ value near 0.90 and low error metrics, the Extreme Gradient Boosting (XGBoost) algorithm showed the best predictive ability out of the six machine learning models created. Its capacity to recognize complex nonlinear patterns and apply regularization to lessen overfitting accounts for its impressive performance.
4. The most important factor affecting surface roughness, according to feature importance analysis is feed rate (importance score > 1.75). While depth of cut emerged as the second most significant feature, a comprehensive understanding of surface roughness requires acknowledging the combined influence of all the three features.

Combining experimental validation with data-driven modelling provides a robust methodology for predicting surface finish and optimizing grinding conditions. This integrated approach holds strong potential for advancing precision manufacturing of titanium alloy components in high-performance sectors.This study was limited to dry grinding of Ti-6Al-4 V using only two abrasive wheels (Al₂O₃ and SiC) and focused solely on surface roughness prediction. Future work should explore coolant-assisted grinding, incorporate additional response variables such as tool wear and residual stress, and extend the machine learning models to broader datasets and materials for enhanced industrial applicability.

## Supplementary Information

Below is the link to the electronic supplementary material.

Supplementary Material 1

Supplementary Material 2

## Data Availability

The datasets generated and/or analysed during the current study are available from the corresponding author upon reasonable request.
