# Supplementary material for: Prediction of the surface roughness of Ti-6Al-4 V alloy during surface grinding using machine learning models
Source: Sci Rep. 2026 Jun 1;16:25541. doi: 10.1038/s41598-026-53166-3 (PMC13478160; doi:10.1038/s41598-026-53166-3)
Supplement: Supplementary file 1 [file 41598_2026_53166_MOESM1_ESM.pdf]

# SUPPLEMENTARY MATERIAL

## 1 MACHINE SPECIFICATIONS

| | |
|---|---|
| Manufacturer | Bhurji Machine Tools |
| Model | AH-630 |
| Working Surface of Table | 600 mm x 300 mm |
| Max. Table Travel | 625 mm x 310 mm |
| Max Dist. Btw. Spindle Centre and Table | 465 mm |
| Wheel Speed | 2800 RPM (29.32 m/s) |
| Wheel Dimensions | (200 x 19 x 31.75) mm |
| Spindle Motor | 3 Phase, 1.5 HP |
| Table Speed | 5-20 m/min |
| Auto Cross Feed Increment | 0.3 – 5.0 mm |
| Cross Feed on Hand Wheel | 5 mm/rev |

## 2 SURFACE ROUGHNESS TESTER SPECIFICATIONS

| | |
|---|---|
| Manufacturer | Mitutoyo |
| Model | SJ-410 (178-583-11A) |
| Measuring Speed | 0.05, 0.1, 0.2, 0.5 mm/s |
| Returning Speed | 0.5, 1, 2 mm/s |
| Detector Z-Range | 8, 80, 800 µm |
| Detector Z-Range Resolution | 0.000125, 0.00125, 0.0125 µm |
| Detector Type (Measuring Principle) | Differential Inductance |
| Skid Radius of Curvature | 40 mm |
| Range X-Axis | 50 mm |
| Measuring Force | 0.75 mN |
| Stylus Tip | Material: Diamond<br>Angle: 60°<br>Tip Radius: 2µm |

# 3 HYPERPARAMETER LIST

## 3.1 Decision Tree

| **Hyperparameter** | **Value** |
|---|---|
| ccp_alpha | 0.0 |
| criterion | squared_error |
| min_impurity_decrease | 0.0 |
| min_samples_leaf | 1 |
| min_samples_split | 2 |
| min_weight_fraction_leaf | 0.0 |
| random_state | 42 |
| splitter | best |

## 3.2 Random Forest

| **Hyperparameter** | **Value** |
|---|---|
| bootstrap | True |
| ccp_alpha | 0.0 |
| criterion | squared_error |
| max_features | auto |
| min_impurity_decrease | 0.0 |
| min_samples_leaf | 1 |
| min_samples_split | 2 |
| min_weight_fraction_leaf | 0.0 |
| n_estimators | 100 |
| oob_score | False |
| random_state | 42 |
| verbose | 0 |
| warm_start | False |

## 3.3 Gradient Boost

| **Hyperparameter** | **Value** |
| --- | --- |
| alpha | 0.9 |
| ccp_alpha | 0.0 |
| criterion | friedman_mse |
| learning_rate | 0.1 |
| loss | squared_error |
| max_depth | 3 |
| min_impurity_decrease | 0.0 |
| min_samples_leaf | 1 |
| min_samples_split | 2 |
| min_weight_fraction_leaf | 0.0 |
| n_estimators | 100 |
| random_state | 42 |
| subsample | 1.0 |
| tol | 0.0001 |
| validation_fraction | 0.1 |
| verbose | 0 |
| warm_start | False |

## 3.4 Extreme Gradient Boost

| **Hyperparameter** | **Value** |
| --- | --- |
| objective | reg:squarederror |
| base_score | 0.5 |
| booster | gbtree |
| colsample_bylevel | 1 |
| colsample_bynode | 1 |
| colsample_bytree | 1 |
| gamma | 0 |
| gpu_id | -1 |

| | |
|---|---|
| grow_policy | depthwise |
| learning_rate | 0.300000012 |
| max_bin | 256 |
| max_cat_to_onehot | 4 |
| max_delta_step | 0 |
| max_depth | 6 |
| max_leaves | 0 |
| min_child_weight | 1 |
| monotone_constraints | () |
| n_estimators | 100 |
| n_jobs | 0 |
| num_parallel_tree | 1 |
| predictor | auto |
| random_state | 42 |
| reg_alpha | 0 |
| reg_lambda | 1 |
| sampling_method | uniform |
| scale_pos_weight | 1 |
| subsample | 1 |
| tree_method | exact |
| validate_parameters | 1 |
| verbosity | 0 |

## 3.5 Linear Regression

| **Hyperparameter** | **Value** |
|---|---|
| copy_X | True |
| fit_intercept | True |
| positive | False |

## 3.6 Polynomial Regression

| Hyperparameter | Value |
|---|---|
| steps | [('polynomialfeatures', 'PolynomialFeatures(degree=3)'), ('linearregression', 'LinearRegression()')] |
| verbose | False |
| polynomialfeatures | PolynomialFeatures(degree=3) |
| linearregression | LinearRegression() |
| polynomialfeatures__degree | 3 |
| polynomialfeatures__include_bias | True |
| polynomialfeatures__interaction_only | False |
| polynomialfeatures__order | C |
| linearregression__copy_X | True |
| linearregression__fit_intercept | True |
| linearregression__positive | False |
