# Supplementary material for: Prediction of the surface roughness of Ti-6Al-4 V alloy during surface grinding using machine learning models
Source: Sci Rep. 2026 Jun 1;16:25541. doi: 10.1038/s41598-026-53166-3 (PMC13478160; doi:10.1038/s41598-026-53166-3)
Supplement: Supplementary file 2 [file 41598_2026_53166_MOESM2_ESM.pdf]

SUPPLEMENTARY MATERIAL

1 MACHINE SPECIFICATIONS

Manufacturer	Bhurji Machine Tools
Model	AH-630
Working Surface of Table	600 mm x 300 mm
Max. Table Travel	625 mm x 310 mm
Max Dist. Btw. Spindle Centre and Table	465 mm
Wheel Speed	2800 RPM (29.32 m/s)
Wheel Dimensions	(200 x 19 x 31.75) mm
Spindle Motor	3 Phase, 1.5 HP
Table Speed	5-20 m/min
Auto Cross Feed Increment	0.3 – 5.0 mm
Cross Feed on Hand Wheel	5 mm/rev

2 SURFACE ROUGHNESS TESTER SPECIFICATIONS

Manufacturer	Mitutoyo
Model	SJ-410 (178-583-11A)
Measuring Speed	0.05, 0.1, 0.2, 0.5 mm/s
Returning Speed	0.5, 1, 2 mm/s
Detector Z-Range	8, 80, 800 µm
Detector Z-Range Resolution	0.000125, 0.00125, 0.0125 µm
Detector Type (Measuring Principle)	Differential Inductance
Skid Radius of Curvature	40 mm
Range X-Axis	50 mm
Measuring Force	0.75 mN
Stylus Tip	Material: Diamond<br>Angle: 60°<br>Tip Radius: 2µm

3 HYPERPARAMETER LIST

3.1 Decision Tree

**Hyperparameter**	**Value**
ccp_alpha	0.0
criterion	squared_error
min_impurity_decrease	0.0
min_samples_leaf	1
min_samples_split	2
min_weight_fraction_leaf	0.0
random_state	42
splitter	best

3.2 Random Forest

**Hyperparameter**	**Value**
bootstrap	True
ccp_alpha	0.0
criterion	squared_error
max_features	auto
min_impurity_decrease	0.0
min_samples_leaf	1
min_samples_split	2
min_weight_fraction_leaf	0.0
n_estimators	100
oob_score	False
random_state	42
verbose	0
warm_start	False

3.3 Gradient Boost

**Hyperparameter**	**Value**
alpha	0.9
ccp_alpha	0.0
criterion	friedman_mse
learning_rate	0.1
loss	squared_error
max_depth	3
min_impurity_decrease	0.0
min_samples_leaf	1
min_samples_split	2
min_weight_fraction_leaf	0.0
n_estimators	100
random_state	42
subsample	1.0
tol	0.0001
validation_fraction	0.1
verbose	0
warm_start	False

3.4 Extreme Gradient Boost

**Hyperparameter**	**Value**
objective	reg:squarederror
base_score	0.5
booster	gbtree
colsample_bylevel	1
colsample_bynode	1
colsample_bytree	1
gamma	0
gpu_id	-1

grow_policy	depthwise
learning_rate	0.300000012
max_bin	256
max_cat_to_onehot	4
max_delta_step	0
max_depth	6
max_leaves	0
min_child_weight	1
monotone_constraints	()
n_estimators	100
n_jobs	0
num_parallel_tree	1
predictor	auto
random_state	42
reg_alpha	0
reg_lambda	1
sampling_method	uniform
scale_pos_weight	1
subsample	1
tree_method	exact
validate_parameters	1
verbosity	0

3.5 Linear Regression

**Hyperparameter**	**Value**
copy_X	True
fit_intercept	True
positive	False

3.6 Polynomial Regression

**Hyperparameter**	**Value**
steps	[('polynomialfeatures', 'PolynomialFeatures(degree=3)'), ('linearregression', 'LinearRegression()')]
verbose	False
polynomialfeatures	PolynomialFeatures(degree=3)
linearregression	LinearRegression()
polynomialfeatures__degree	3
polynomialfeatures__include_bias	True
polynomialfeatures__interaction_only	False
polynomialfeatures__order	C
linearregression__copy_X	True
linearregression__fit_intercept	True
linearregression__positive	False
